# An Improved Method for Spot Position Detection of a Laser Tracking and Positioning System Based on a Four-Quadrant Detector

**DOI:** 10.3390/s19214722

**Published:** 2019-10-30

**Authors:** Wugang Zhang, Wei Guo, Chuanwei Zhang, Shuanfeng Zhao

**Affiliations:** School of Mechanical Engineering, Xi’an University of Science and Technology, Xi’an 710054, China; guow@xust.edu.cn (W.G.); zhangcw@xust.edu.cn (C.Z.); zsf@xust.edu.cn (S.Z.)

**Keywords:** laser tracking and positioning, four-quadrant detector, piecewise low-order 18 polynomial fitting, Kalman filter

## Abstract

For the laser tracking and positioning system of a moving target using a four-quadrant detector, the accuracy of laser spot position detection has a serious impact on the tracking performance of the system. For moving target tracking, the traditional spot position detection method of a four-quadrant detector cannot give better consideration to both detection accuracy and operation speed. In view of this, an improved method based on piecewise low-order polynomial least squares fitting and a Kalman filter is proposed. Firstly, the tracking and positioning mathematical model of the system is created, and the experimental device is established. Then, the shortcomings of traditional methods are analyzed, and the improved method and the real-time tracking and positioning algorithm of the system are studied. Finally, through the experiment, the system operation effects are compared and analyzed before and after the improvement. The experimental results of system dynamic tracking show that, the least squares fitting of the experimental data using a 5-segment and quadratic polynomial can achieve better results. By using the improved method, the maximum tracking distance of a moving object is increased from 12 m to more than 30 m. At a distance of 7.5 m, the maximum tracking speed can reach 2.11 m/s, and the root mean square error (RMSE) of the position is less than 4.59 mm. At 15.5 m, the maximum tracking speed is 2.04 m/s and the RMSE is less than 5.42 mm. Additionally, at 23.5 m, it is 1.13 m/s and 5.71 mm.

## 1. Introduction

Laser tracking technology has a wide range of applications in the fields of laser guidance [1], space optical communication [2,3], optical tweezers [4], and large size precision measurement [5,6,7]. Compared with other non-contact positioning methods, such as wireless sensor networks [8] and inertial navigation positioning [9], laser tracking and positioning has the advantages of high accuracy and strong environmental adaptability. At present, laser trackers have been widely used in moving target tracking; its ranging accuracy has exceeded the sub-micron scale, and its measuring distance up to hundreds of meters. However, due to its complex structure, high precision, and large volume, the laser tracker has higher requirements for the environment, which is not suitable for general precision requirements and harsh environments, such as coal mines, tunnels, and large storage, etc. In addition, due to the high price, it is not suitable to build a measurement network for multiple laser trackers.

In this paper, a low-cost device for moving target tracking based on a two-dimensional (2D) galvanometer is studied, which is called a laser tracking and positioning system (LTPS). It uses a four-quadrant detector (4-QD) to get the position information of the laser spot that has returned from the moving target, and adjusts a 2D galvanometer to change laser direction, and then the target tracking function is realized. For the whole system, the accuracy of spot position detection directly determines the tracking and positioning performance. In order to improve the detection accuracy of the 4-QD, scholars have done a lot of research on the algorithm of spot position detection. Chen [10] and Vo [11] used the polynomial fitting algorithm to study the nonlinear relationship between the detection signal and the spot position. Through experiments, the relationship between the spot position and the output voltages of 4-QD was obtained, and the curve was fitted with a high-order polynomial. Salmanpour [12] proposed a nonlinear correction method based on a BP (Back Propagation) neural network for 4-QD to calculate the spot position. Lee [13] studied the method of calculating the spot position of Gaussian energy distribution, and found that for different spot diameters, the sensitivity of the detector increases with the decrease of spot radius. Lu [14] explored the detection method of an annular light spot and studied it using the annular segmentation integral method. Li and Xu [3] used cosine modulated light and a cyclic cross correlation method to detect the spot position, and obtained the accurate signal amplitude and improved the precision. Wu [15] used the Boltzmann function to construct the spot position curve to improve detection accuracy. In addition, Xiao [16,17] and other scholars have made in-depth studies on the theory and method of multi-sensor information fusion. These methods can also be a feasible way to improve the spot position accuracy of a laser tracking system.

Most of the existing methods use the idealized model and treat the shape of the spot as an approximate circle, and regard the energy distribution as a uniform distribution or Gaussian distribution. Under this assumption, the spot position detection methods are studied by analytical methods. For the LTPS presented in this paper, the shape and energy distribution of light spots are irregular, and there are many factors affecting the accuracy of spot position detection. Therefore, it is difficult to establish an accurate model using traditional analytical methods.

For LTPS, the factors that affect the accuracy of spot position detection are mainly divided into two categories. One is the inherent errors of the system, the other includes random errors. The inherent errors mainly include the model error of spot position detection, the four quadrant inconsistency error, the optical adjustment error, the optical element distortion error, and so on. The random errors mainly include the conversion and amplification noise, the background noise, the sampling errors of output signals and other noise caused by speckle energy fluctuation, electromagnetic interference, temperature drift, dust and atmospheric turbulence etc. It is not easy to use analytical methods to establish the mathematical model to study spot position detection. Therefore, experimental analysis methods will be used to study the improving method. In order to establish the spot position detection model with a simple algorithm and fast processing speed, the piecewise low-order polynomial fitting algorithm is adopted to fit the experimental data, and the fitting curve between the output voltages of the 4-QD and the spot position satisfying the tracking accuracy of the system is obtained, which can be used as the improved model to compensate for the inherent errors. For the random errors, the real-time Kalman filtering algorithm is proposed to filter the output voltages of the 4-QD to reduce the influence of various random errors. The improved method proposed in this paper takes the output values of the Kalman filter as the best estimate of the true output voltages of the 4-QD. Then, the filtered voltages are put into the low-order polynomial fitting curves to obtain the high-precision spot position values. The system takes the spot position value as the position feedback signal, controls the 2D galvanometer, adjusts the angle of laser injection, and realizes the tracking and positioning of moving objects.

In this paper, an improved method for spot position detection of LTPS is presented. In Section 2, the principle of LTPS, the mathematical model of tracking and positioning, and the experimental system are introduced. In Section 3, the traditional methods of spot position detection are described. In Section 4, the improved method based on spot position detection using piecewise low-order polynomial least squares fitting and the Kalman filter are introduced in detail. In Section 4, the results of moving target tracking and positioning experiments are analyzed. Finally, the conclusion is given in Section 5.

## 2. The Principle and Experimental Equipment of the System

### 2.1. Composition Principle of the LTPS

**Figure 1:**
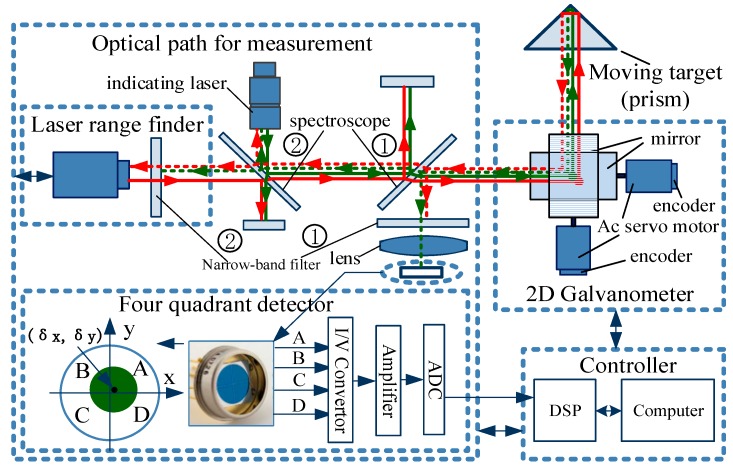
Composition principle of the laser tracking and positioning system (LTPS).

The composition principle of the LTPS is shown in Figure 1, which is mainly composed of the target prism, measuring the optical path, 2D galvanometer, DSP (Digital Signal Processing), computer, and other components. The prism is fixed on the target body and moves with the target in three-dimensional space. Measuring the optical path is mainly composed of an indicating laser, a laser rangefinder, two groups of spectroscopes, 4-QD, lens, etc., and mainly realizes the functions of laser emission, reflected light receiving, and spot position detection. The high power laser with 520 nm wavelength is used as the indicator laser. Additionally, the laser wavelength of the laser rangefinder is 635 nm. The two-channel laser beams are combined by two groups of spectroscopes, 1 and 2, and are refracted by the 2D galvanometer and projected onto the target prism. The reflected lights return along the original path and enter the 2D galvanometer. Then, they are divided into two lines by Spectroscope 1. The first light passes through Narrow-band filter 1 and lens; only the 520 nm light can enter the surface of 4-QD to generate the signal of spot position detection. The output voltages of the 4-QD are sampled into the DSP controller by the ADC (Analog-to-Digital Convertor) module, and the spot position $\left(x_{\delta },y_{\delta }\right)$ is calculated. The second light passes through Spectroscope 2 and Narrow-band filter 2. Only 635 nm light can enter the laser rangefinder and generate the ranging signal. The rangefinder calculates the target distance and sends it to the system.

The 2D galvanometer is composed of two sets of galvanometers whose axes are perpendicular to each other. Each set of galvanometers is composed of a servo motor, driver, encoder, and reflector. The servo motors of the 2D galvanometer are controlled by the DSP controller according to the spot position value $\left(x_{\delta },y_{\delta }\right)$ of the 4-QD, to ensure that the indicating laser always aims at the center of the prism. The computer system collects the target distance of the laser rangefinder and two rotation angles of the 2D galvanometer (horizontal angle θx and pitch angle θz of the laser beam) in real time and calculates the position of the moving target.

### 2.2. The Mathematical Model of Tracking and Positioning

**Figure 2:**
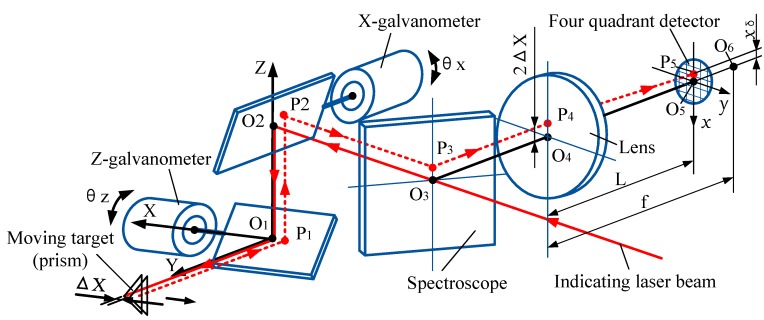
Schematic diagram of laser tracking.

Figure 2 shows the schematic diagram of laser tracking of the LTPS. The two rotating axes of the galvanometer are X and Z, and the two mirrors rotate under the drive of the motors. In the initial position of the 2D galvanometer, the two mirrors are respectively 45° from the mounting base, and the angles of the two mirrors are θx = 0 and θz = 0. In the target moving space, the measurement coordinate system O_1_ (x,y,z) is established, and the origin is located at the center of the Z-axis galvanometer.

The indicating laser beam is projected through the center of the spectroscope O_3_ at the center of the X-galvanometer O_2_. After reflection by the galvanometer, it is projected to the center of the target prism with the vertex on the Y-axis. The reflected light returns along the original path and coincides with the position of the incident light in the opposite direction and is refracted by the spectroscope and projected at the center of the lens O_4_. In Figure 2, the focal length of the lens is f, and O_6_ is the focal point. The 4-QD is located in front of the focal plane, and the distance between it and the lens is L. The returned beam is converged through the lens and projected onto the center O_5_ of the 4-QD.

For a short period of time, when the target prism moves along the ΔX distance in the X-direction, the reflected light will no longer coincide with the incident light, but is still parallel with it, deviating from it 2ΔX distance, and projecting at point P1. Then the reflected light passes through P_2_ and P_3_ to the point P_4_, which is converged through the lens and projected to the point P_5_ on the surface of the 4-QD. Line O_5_P_5_ is denoted as $x_{\delta }$, which is the spot position in the X-direction. According to the known parameters of the system, the relationship between the moving distance ΔX and the detection value $x_{\delta }$ of the 4-QD can be obtained, which can be expressed as follows:

(1) $$\Delta X=\frac{f}{2(f-L)}x_{\delta }=a\cdot x_{\delta }.$$

In the formula, f is the focal length of the lens, L is the distance between the lens and the 4-QD, and a is a constant for a given system and a=f/2(f−L).

Similarly, when the target moves parallel to the *Z* direction, the relationship between the moving distance Δ*Z* and the detection value $y_{\delta }$ is as follows:

(2) $$\Delta Z=\frac{f}{2(f-L)}y_{\delta }=a\cdot y_{\delta }.$$

In order to achieve target tracking, the system needs to calculate the target moving amount (Δ*X*, Δ*Z*) according to the spot position value $\left(x_{\delta },y_{\delta }\right)$, then calculate the adjusting angles of the 2D galvanometer (*θx*, *θz*), and adjust the rotation of the galvanometer servo motor, until the value of spot position detection is (0,0) and the indicator laser is re-projected into the center of the target prism. The relationship between the target moving distance (Δ*X*, Δ*Z*) and the rotation angles of the 2D galvanometer can be described as in [18].

(3) $$\{\begin{array}{l} \theta_{x}=\frac{1}{2}arc\sin\frac{\Delta X}{D-l}\\ \theta_{z}=\frac{1}{2}arc\sin\frac{\Delta Z}{\sqrt{{\left(D-l\right)}^{2}-\Delta X^{2}}-e} \end{array}$$

In the formula, e is the distance between two axes of the 2D galvanometer; D is the distance between the prism and the laser rangefinder; l is the distance between the laser rangefinder and the center of the *X*-axis galvanometer O_2_. For a given system, e and l are known parameters.

From Equations (1)–(3), the tracking mathematical model of LTPS can be established as follows:

(4) $$\{\begin{array}{l} \theta_{x}=\frac{1}{2}arc\sin\frac{ax_{\delta }}{D-l}\\ \theta_{z}=\frac{1}{2}arc\sin\frac{ay_{\delta }}{\sqrt{{\left(D-l\right)}^{2}-a^{2}x_{\delta }{}^{2}}-e} \end{array}.$$

According to the angles (*θx*, *θz*) of the 2D galvanometer and the target distance D, the positioning mathematical model of LTPS can be obtained as follows [18]:

(5) $$\left[\begin{array}{c} x\\ y\\ z \end{array}\right]=(D-l)\cdot \left[\begin{array}{c} \cos2\theta_{x}\cdot \sin2\theta_{z}\\ \cos2\theta_{x}\cdot \cos2\theta_{z}\\ \sin2\theta_{x} \end{array}\right]+\left[\begin{array}{c} -e\cdot \sin2\theta_{z}\\ -e\cdot \cos2\theta_{z}\\ 0 \end{array}\right]$$

where (x,y,z) is the current coordinate of the moving target.

### 2.3. System Construction and Experiment Device

**Figure 3:**
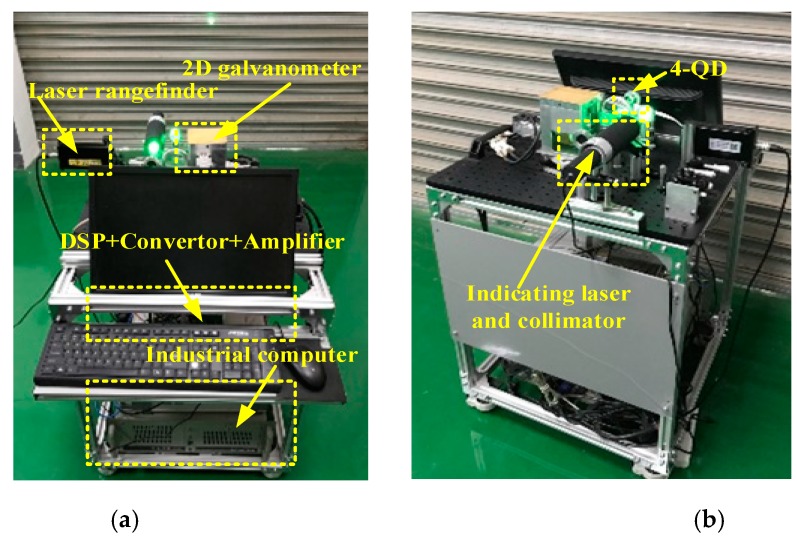
Experimental setup of LTPS: (**a**) forward-looking; (**b**) back view.

The experimental equipment of LTPS is shown in Figure 3. It is mainly composed of an indicating laser and optical collimator, 4-QD, 2D galvanometer, DSP controller, amplifier conversion card, movable optical platform, industrial computer, and so on. It uses the SKD-100D laser rangefinder (Sankoe, Xi’an, China), with a laser wavelength of 635 nm, maximum measuring distance of 100 m, and a measurement accuracy of ±1 mm. The indicated laser wavelength is 520 nm, the divergence angle of optical platform is less than 0.02 mrad, and the outgoing light diameter is 30 mm. The system uses a 4-QD of type QP50-6 (First Sensor, Berlin, Germany), with a diameter of 7.8 mm and a dead zone of 42 μm. Each of the galvanometer units of the 2D galvanometer contains a 21-bit encoder and an AC (Alternating Current) servo motor. Both galvanometers are controlled by the DSP controller (Chip model: TMS320F28335). The measuring range of the galvanometer is $\pm 14^{{}^{\circ}}$ of the X-scanner and $\pm 16^{{}^{\circ}}$ of the Z-scanner. 

## 3. Spot Position Detection Method

### 3.1. Spot Position Detection

**Figure 4:**
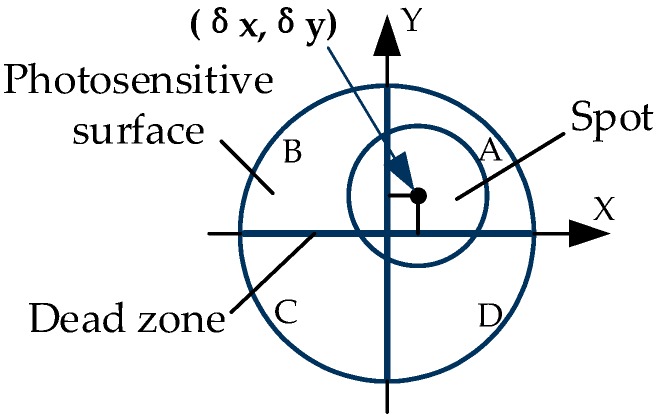
The principle of the four-quadrant detector (4-QD).

The principle of the 4-QD is shown in Figure 4. The photosensitive surface of the detector is divided into four regions (A, B, C, D) with the same shape and area by the dead zone. When the detector works, the laser beam reflected by the target prism is concentrated on the photosensitive surface of the 4-QD through the optical system, and the centroid coordinate of the spot is (δx,δy). At this time, the energy received in each quadrant of the 4-QD is $I_{n}(n=A,B,C,D)$. When the spot moves on the photosensitive surface, the energy received by each quadrant will change correspondingly with the displacement, leading to the change of the output photocurrent. The centroid coordinate of the spot can be calculated by the energy in each quadrant according to Equation (6) [19]:

(6) $$\{\begin{array}{c} x_{\delta }\approx \delta x=k\sigma_{x}=k\frac{I_{A}+I_{D}-(I_{B}+I_{C})}{I_{A}+I_{B}+I_{C}+I_{D}}\\ y_{\delta }\approx \delta y=k\sigma_{y}=k\frac{I_{A}+I_{B}-(I_{C}+I_{D})}{I_{A}+I_{B}+I_{C}+I_{D}} \end{array}$$

where $\left(x_{\delta },y_{\delta }\right)$ are the true positions of the spot; $\left(\delta_{x},\delta_{y}\right)$ are the centroid coordinates of the spot; k is the proportional coefficient related to the spot; $\sigma_{x}$ and $\sigma_{y}$ are the normalized values calculated for the spot center position, and $\sigma_{x},\sigma_{y}\in [-1,1]$. 

### 3.2. Traditional Detection Method

The centroid coordinate of the spot is related to energy distribution of the spot. With different energy distributions, the centroid coordinate of the spot calculated according to Equation (6) will have different errors.

(1) Uniform distribution of spot energy

Sometimes, in order to simplify the problem, the spot energy distribution is considered to be uniform. In this case, the photocurrent in each quadrant is proportional to the received energy of the spot. The position of the spot can be estimated by the output voltages of the four quadrants’ photodiodes according to Equation (7) [20].

(7) $$\{\begin{array}{c} x_{\delta }\approx \delta x=k\frac{I_{A}+I_{D}-(I_{B}+I_{C})}{I_{A}+I_{B}+I_{C}+I_{D}}=k\frac{\left(U_{A}+U_{D})-(U_{B}+U_{C}\right)}{U_{A}+U_{B}+U_{C}+U_{D}}\\ y_{\delta }\approx \delta y=k\frac{I_{A}+I_{B}-(I_{C}+I_{D})}{I_{A}+I_{B}+I_{C}+I_{D}}=k\frac{\left(U_{A}+U_{B})-(U_{C}+U_{D}\right)}{U_{A}+U_{B}+U_{C}+U_{D}} \end{array}$$

In the formula, $U_{n}(n=A,B,C,D)$ is the output voltage of the four quadrants’ photodiodes, which is proportional to the received energy of each quadrant.

This method only uses simple algebraic operations and has less computational complexity. It is easy to implement in microprocessors and has a faster speed. However, because the spot energy does not obey uniform distribution strictly, the accuracy of the spot position calculated by this method is not high.

(2) Gaussian distribution of energy

Compared with the uniform distribution, the spot energy is closer to the Gaussian distribution. The center of the spot has the strongest energy, which is circular and diffuses outwards, and gradually weakens when leaving the center. If the coordinate of the center is denoted by $\left(\delta x_{0},\;\delta y_{0}\right)$, and the light intensity value on the photosensitive surface is denoted by I(δx,δy), then the probability density function of the spot energy equivalent to the 2D Gauss distribution, as is shown in Equation (8) [21].

(8) $$I(\delta x,\delta y)=\frac{I_{0}}{2\pi \sigma^{2}}\exp[-\frac{{\left(\delta x-\delta x_{0}\right)}^{2}+{\left(\delta y-\delta y_{0}\right)}^{2}}{2\sigma^{2}}]$$

In the formula, $I_{0}$ is the total energy of the spot and σ is the beam waist radius of laser energy distribution.

For the Gaussian spot, more than 90% of its energy is concentrated in the circle with a radius of 2σ. Therefore, the total energy of the spot with Gaussian energy distribution can be approximated by an infinite integral, as shown in Equation (9).

(9) $$I_{0}=I_{A}+I_{B}+I_{C}+I_{D}=\frac{I_{0}}{2\pi \sigma^{2}}\int_{-\infty }^{\infty }\int_{-\infty }^{\infty }\exp[-\frac{{\left(\delta x-\delta x_{0}\right)}^{2}+{\left(\delta y-\delta y_{0}\right)}^{2}}{2\sigma^{2}}]d\delta xd\delta y$$

The radius of the photosensitive surface of the 4-QD is R. Assuming that the light energy outside the photosensitive surface of the spot is ignored, the upper and lower limits of the integral can be set to infinity, then the following formula can be obtained [22]:

(10) $$I_{A}=\frac{I_{0}}{2\pi \sigma^{2}}\int_{0}^{\infty }\int_{0}^{\infty }\exp(-\frac{{\left(\delta x-\delta x_{0}\right)}^{2}+{\left(\delta y-\delta y_{0}\right)}^{2}}{2\sigma^{2}})\;d\delta xd\delta y$$

(11) $$I_{B}=\frac{I_{0}}{2\pi \sigma^{2}}\int_{0}^{\infty }\int_{-\infty }^{0}\exp(-\frac{{\left(\delta x-\delta x_{0}\right)}^{2}+{\left(\delta y-\delta y_{0}\right)}^{2}}{2\sigma^{2}})\;d\delta xd\delta y$$

(12) $$I_{C}=\frac{I_{0}}{2\pi \sigma^{2}}\int_{-\infty }^{0}\int_{-\infty }^{0}\exp(-\frac{{\left(\delta x-\delta x_{0}\right)}^{2}+{\left(\delta y-\delta y_{0}\right)}^{2}}{2\sigma^{2}})\;d\delta xd\delta y$$

(13) $$I_{D}=\frac{I_{0}}{2\pi \sigma^{2}}\int_{-\infty }^{0}\int_{0}^{\infty }\exp(-\frac{{\left(\delta x-\delta x_{0}\right)}^{2}+{\left(\delta y-\delta y_{0}\right)}^{2}}{2\sigma^{2}})\;d\delta xd\delta y.$$

Equations (10)–(13) are introduced into Equation (6), and the formula for calculating the spot position with Gaussian distribution is obtained. The Gaussian distribution method can obtain better accuracy than uniform distribution, but the calculation time is longer due to the complexity of integral calculation.

### 3.3. Spot Position Detection of the LTPS

**Figure 5:**
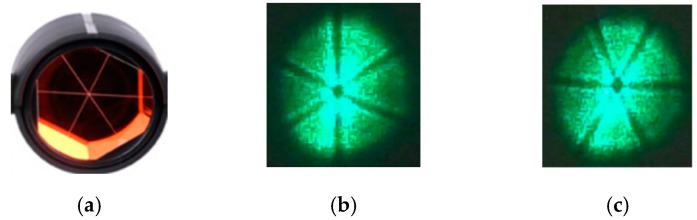
Target prisms and reflective spots. (**a**) the prism; (**b**) reflected light spot 1; (**c**) reflected light spot 2.

For the LTPS described in this paper, the prism was used as the target reflector, as shown in Figure 5a. When an incident laser is reflected by a prism, its shape and energy distribution will change. The shape of the spot reaching the surface of the 4-QD is shown in Figure 5b,c.

As can be seen from Figure 5, due to the reduced reflection effect of the vertex and edge of the prism, there are six strips of dark areas in the center of the reflected light, the shape of which coincides with the shape of the prism. Thus, the distribution of spot energy is not uniform and no longer conforms to the Gaussian distribution. When the prism moves with the target, these strip dark areas fluctuate between the positions shown in Figure 5b,c. 

**Figure 6:**
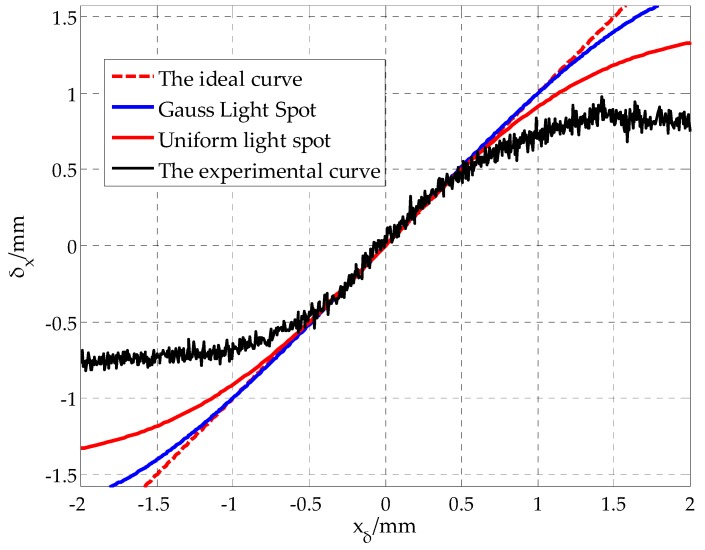
Comparison between the simulated curve and the actual curve in the X-direction.

Taking the spot position in X-direction as an example, for a given theoretical $x_{\delta }$ value, the spot position $\delta_{x}$ is calculated by uniform distribution and Gaussian distribution, respectively, and then the two simulation curves of $x_{\delta }-\delta_{x}$ are drawn, as shown in the red and blue curves in Figure 6.

Data acquisition experiments were carried out, and by moving the 4-QD on the precise displacement platform, the output voltage of the detector $\left(U_{A}^{},U_{B}^{},U_{C}^{},U_{D}^{}\right)$ and the actual displacement of the spot $x_{\delta }$ were collected synchronously. The calculated value of the spot position $\delta_{x}$ was obtained by introducing type (6), then the experimental curve $x_{\delta }-\delta_{x}$ was obtained, as shown in the black curve in Figure 6.

Through comparative analysis, if the spot is regarded as an ideal circular spot, the strip dark areas are neglected, and the effects of various inherent errors and random errors of the system are not considered; there is an obvious error between the spot position calculated by the traditional method and the actual spot position. Similar problems exist in the Y-direction of the 4-QD as in the X-direction. Therefore, in order to get the spot position with high accuracy, the influence of spot shape distortion, system inherent errors, and random errors should be considered comprehensively, and the traditional methods should be improved.

## 4. Improved Method for Spot Position Detection

### 4.1. The Improved Method

Because of the existence of inherent errors and random errors, the traditional calculation method of Equation (7) has a large error. The improved method takes measures to reduce the impact of the two kinds of errors. Because the experimental data $\left\{x_{\delta }:\delta_{x}\right\}$ has implied various spot position errors caused by the spot shape, strip dark area, and various inherent errors, the analytical equation between $x_{\delta }$ and $\delta_{x}$ can be established by fitting the experimental data $\left\{x_{\delta }:\delta_{x}\right\}$, and the fitting curve can be used as a high-precision spot position detection model. For the output voltage fluctuation caused by random errors, a Kalman filter can be used to obtain the best estimation of the output voltage from a series of data with noise when the variance is known, so as to reduce the noise and improve the detection accuracy.

**Figure 7:**
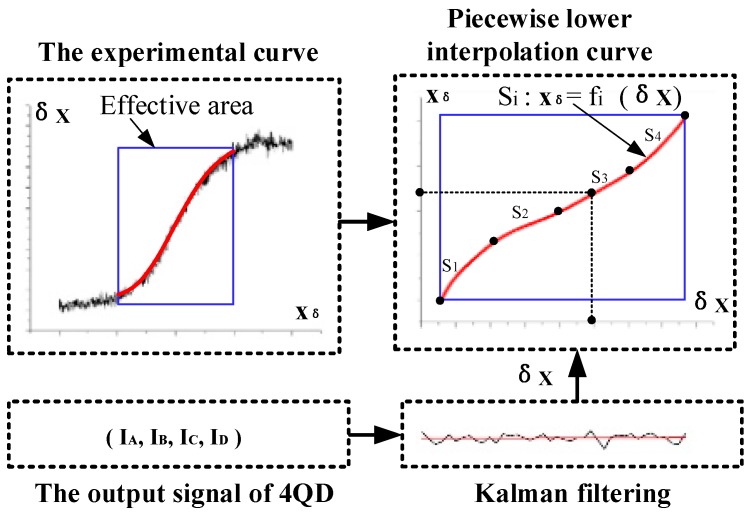
Schematic diagram of the improved method.

The schematic diagram of the improved method for spot position detection is shown in Figure 7. The experimental curve $x_{\delta }-\delta_{x}$ of the 4-QD was fitted in the effective detection area. Because the high order curve is not suitable for real-time processing by the microprocessor, the piecewise low-order polynomial least squares fitting was used to fit the experimental data $\left\{x_{\delta }:\delta_{x}\right\}$, and then the piecewise curve fitting equation $S_{i}:x_{\delta }=f(\delta_{X})\;(i=1,2,3,\cdots )$ was obtained.

In each tracking period, the system sampled the four-channel voltages of the detector for n times to obtain the sampling value ${\left(U_{A}^{},U_{B}^{},U_{C}^{},U_{D}^{}\right)}_{j}(j=1,2,\cdots n)$. The Kalman filtering algorithm was adopted to filter the output voltages, and then Equation (6) was used to obtain the calculation value $\delta_{x}^{i}(i=1,2\cdots n)$ of the spot position. Putting $\delta_{x}$ into the piecewise curve $S_{i}:x_{\delta }=f(\delta_{x})\;(i=1,2,3,\cdots )$ obtains the high precision spot position value $x_{\delta }$. Since the X-direction and Y-direction of the 4-QD are independent from each other, the improved method in the Y-direction is the same as that in X. 

### 4.2. Piecewise Low-Order Polynomial Least Squares Fitting

From the experimental curves, it can be seen that the actual position of the spot has a non-linear relationship with the calculated value of the spot. If the whole curve is fitted, the degree of the fitting curves satisfying the accuracy is higher, which is not suitable for microprocessor processing. In this paper, piecewise low-order polynomial least squares fitting was used to reduce the degree of the polynomial by increasing the number of sections, so as to ensure the fitting accuracy. 

For the experimental data $\left\{\delta_{x}:x_{\delta }\right\}$, it can be divided into several parts. For any part, let the data set be $\left(\delta_{x\;i},x_{\delta \;i})\;(i=1,2,\ldots ,m\right)$, and the process of curve fitting is to find the least squares fitting function $f(\delta_{xi})\in \Phi$ in the polynomial function Φ, so as to minimize the sum of square error $r_{i}=f(\delta_{x\;i})-x_{\delta \;}{}_{i}\;(i=1,2,\ldots ,m)$, that is:

(14) $$\sum_{i=0}^{m}r_{i}^{2}=\sum_{i=0}^{m}{\left[f(\delta_{x\;i})-x_{\delta \;}{}_{i}\right]}^{2}=\min.$$

Here, Φ is a function of polynomials of no more than n(n≤m). The upper formula is to find $f(\delta_{xi})=\sum_{k=0}^{m}a_{k}\cdot \delta_{xi}^{k}\in \Phi$, so that:

(15) $$I=\sum_{i=0}^{m}\left[f(\delta_{xi}\right)-x_{\delta }{}_{i}{]}^{2}=\sum_{k=0}^{m}(\sum_{k=0}^{n}a_{k}\delta_{xi}^{k}-x_{\delta }{}_{i}{)}^{2}.$$

The normal equations can be obtained from the above formulas:

(16) $$\left[\begin{array}{cccc} m+1 & \sum_{i=0}^{m}\delta_{xi} & \ldots & \sum_{i=0}^{m}\delta_{xi}^{n}\\ \sum_{i=0}^{m}\delta_{xi} & \sum_{i=0}^{m}\delta_{xi}^{2} & \ldots & \sum_{i=0}^{m}\delta_{xi}^{n+1}\\ \vdots & \vdots & \vdots & \vdots \\ \sum_{i=0}^{m}\delta_{xi}^{n} & \sum_{i=0}^{m}\delta_{xi}^{n+1} & \ldots & \sum_{i=0}^{m}\delta_{xi}^{2n} \end{array}\right]\left[\begin{array}{c} a_{0}\\ a_{1}\\ \vdots \\ a_{n} \end{array}\right]=\left[\begin{array}{c} \sum_{i=0}^{m}x_{\delta }{}_{i}\\ \sum_{i=0}^{m}\delta_{xi}x_{\delta }{}_{i}\\ \vdots \\ \sum_{i=0}^{m}\delta_{xi}^{n}x_{\delta }{}_{i} \end{array}\right].$$

The least squares fitting polynomial $x_{\delta }=f(\delta_{xi})=\sum_{k=0}^{m}a_{k}\cdot \delta_{xi}^{k}$ can be obtained by solving $a_{k}(k=0,1,\ldots ,n)$ from the above equation. The fitting error is:

(17) $$MSE=\frac{1}{m}\cdot \sum_{i=0}^{m}{\left[f(\delta_{Xi})-x_{\delta }{}_{i}\right]}^{2}.$$

In each control cycle of the LTPS, the output voltages ${\left(U_{A}^{},U_{B}^{},U_{C}^{},U_{D}^{}\right)}_{j}(j=1,2,\cdots n)$ of the 4-QD are sampled in DSP, and the spot position $\delta_{x}^{i}(i=1,2\cdots n)$ is calculated according to Equation (6). Then, the spot position $\delta_{x}^{i}(i=1,2\cdots n)$ is introduced into the least squares polynomial function $x_{\delta }=f(\delta_{x})$, and the high precision position value $x_{\delta }$ of the spot in X-direction can be quickly calculated.

### 4.3. Kalman Filtering

The main random factors that affect the output voltages ${\left(U_{A}^{},U_{B}^{},U_{C}^{},U_{D}^{}\right)}_{j}(j=1,2,\cdots n)$ of the 4-QD can be regarded as Gaussian white noise. A Kalman filter was used to filter the output voltages, which can effectively reduce the influence of noise.

The fluctuation of the output voltages of the 4-QD with time can be regarded as a dynamic process, and the state space model as follows is used to describe the state fluctuation of the output voltage of the 4-QD.

State equation:

(18) X(k+1)=ΦX(k)+ΓW(k)

Observation equation:

(19) Z(k+1)=HX(k)+V(k)

where, k is discrete time; $X(k)\in R^{n}$ is the state of detector output signal X at time k; $\Phi \in R^{n\times n}$ is the state transition matrix; Γ is the noise driving matrix; $Z(k)\in R^{m}$ is the observation signal at corresponding time; $H\in R^{m\times n}$ is the observation matrix; $W(k)\in R^{n}$ is the input noise; $V(k)\in R^{m}$ is the observation noise. The random noise W(k) and R(k) that cause the fluctuation of the output signal can be considered as irrelevant white noise whose mean value is zero and variance is Q and R, respectively.

The core of the Kalman filtering algorithm is to use the observation quantity to estimate the state value. It can be divided into prior estimation and posterior estimation, which are respectively called the prediction part and update part [23,24]. Therefore, the algorithm was divided into the following three parts.

Initialization:

(20) $$\hat{X}(0|0)=\mu_{0}$$

(21) $$P(0|0)=P_{0}$$

Prediction part:

(22) $$\hat{X}(k+1|k)=\Phi \hat{X}(k|k)$$

(23) $$P(k+1|k)=\Phi P(k|k)\Phi^{T}+\Gamma Q\Gamma^{T}$$

Update part:

(24) $$\hat{X}(k+1|k+1)=\hat{X}(k+1|k)+K(k+1)\varepsilon (k+1)$$

(25) $$\varepsilon (k+1)=Z(k+1)-H\hat{X}(k+1|k)$$

(26) $$K(k+1)=P(k+1|k)H^{T}{\left[HP(k+1|k)H^{T}+R\right]}^{-1}$$

(27) $$P(k+1|k+1)=[I_{n}-K(k+1)H]P(k+1|k)$$

Here, $\hat{X}(k|k)$ is the system state estimate at the last time; $\hat{X}(k+1|k)$ is the state of time k + 1 predicted according to the state of time k; P(k+1|k) is the predicted covariance value; $\hat{X}(k+1|k)$ is a one-step prediction value of the system state; K(k+1) is the Kalman gain; ε(k+1) Kalman innovation; P(k+1|k+1) is the covariance value at time k + 1.

From the flow chart of the algorithm, it can be seen that the prediction part is to predict the state of the system one-step with the observation of the previous moment, and the update part is to estimate the state of the next moment with the one-step prediction value of the state and the observation value of the current moment, and revise the updated value of the time. The main framework of the Kalman filtering algorithm is a cycle consisting of prior estimation and posterior estimation.

### 4.4. The Controlling Algorithm of the Improved Method

**Figure 8:**
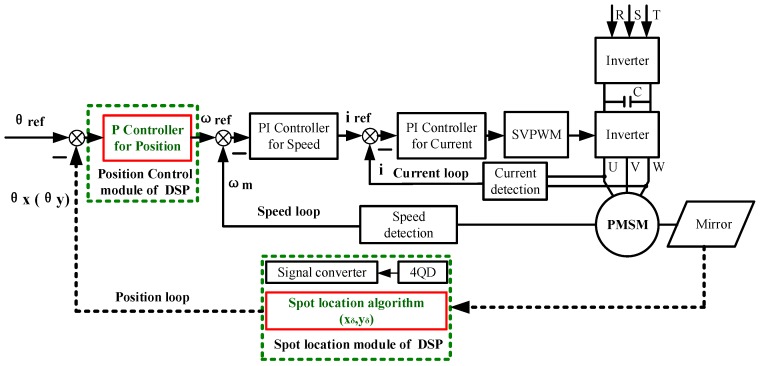
Principle diagram of the controlling algorithm.

A full closed-loop of the AC servo system was adopted to control the 2D galvanometer and to realize real-time tracking of moving objects. The schematic diagram of the control system is shown in Figure 8. In this system, the permanent magnet synchronous motor (PMSM) is used as the AC servo motor, and the space vector pulse width modulation (SVPWM) is used to control the servo motor.

The AC servo motors of each galvanometer adopt three closed-loop control modes, in which the current loop and speed loop adopt PI (Proportion-Integration) control mode and are completed in the servo driver. The position loop adopts proportional control mode, and the angles of the 2D galvanometer (θx, θz) were calculated by DSP accordingly, and will be used as the feedback signal to form the closed-loop control.

The position loop control program was also implemented in DSP, and three tasks were mainly completed in each cycle. The first is to determine which section of the fitting curve the spot is located in, according to the spot position $\left(x_{\delta },y_{\delta }\right)$, and enter the corresponding control program. The second is to calculate the three parameters needed to generate the position command pulse. These parameters include the proportional control coefficient $P_{L}$, the number of pulses N, and the pulse frequency f, which are used to determine the fast responsiveness, tracking quantity, and tracking speed, respectively.

During the position control of the system, the gain of the position loop varies proportionally at different distances. The command pulse parameters of position control are as follows:

Position loop gain:

(28) $$P_{L}=\frac{P_{0}}{L}.$$

In the formula, $P_{L}$ is the position loop gain at the distance *L*; *P*_0_ is the gain of the position loop at the known reference position; *L* is the target distance.

The number of command pulses is calculated as follows:

(29) $$n_{x}=\frac{\theta_{x}}{360{}^{\circ}}\times N$$

(30) $$n_{z}=\frac{\theta_{y}}{360{}^{\circ}}\times N.$$

In the formula, $n_{x}$ is the number of pulses of the *X*-axis galvanometer; *θx* is the angle of the *X*-axis galvanometer; $n_{z}$ is the number of pulses of the *Z*-axis galvanometer; *θy* is the angle adjustment of the *Z*-axis galvanometer; *N* is the number of pulses required for each rotation of the servo motor.

When the spot is at $x_{\delta }$ position, the command pulse frequency is calculated as follows:

(31) $$\omega_{x}=\frac{\omega_{\max}\cdot x_{\delta }}{0.5R}\;(\mathrm{rad}/s)$$

(32) $$f=\frac{\omega_{x}}{2\pi }\cdot N\;(\mathrm{Hz})$$

In the formula, $\omega_{x}$ is the tracking angular velocity; $\omega_{\max}$ is the highest tracking angular velocity of the system (influenced by system hardware, servo system performance, and tracking control algorithm); *R* is the effective photosensitive surface radius of the 4-QD; f is the command pulse frequency.

**Figure 9:**
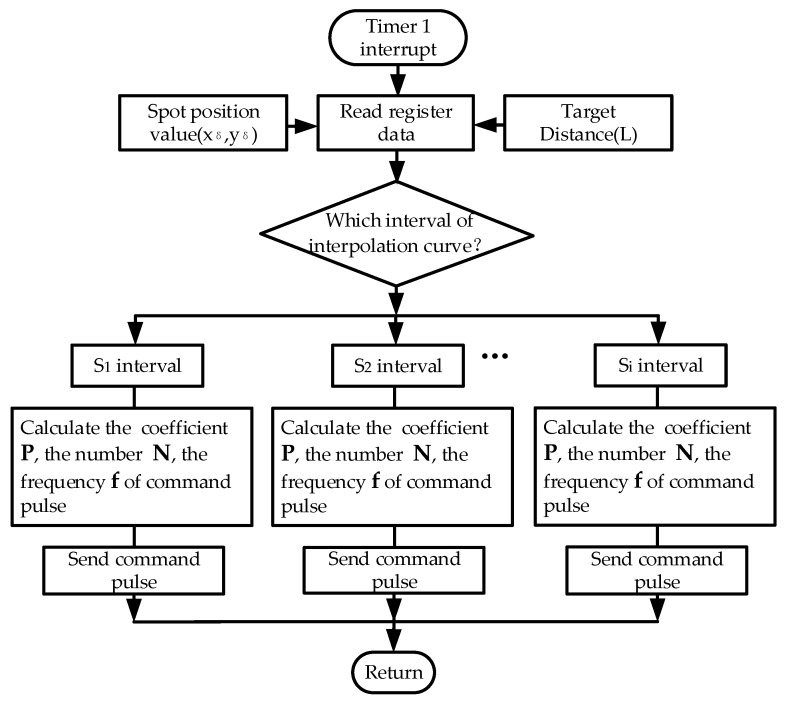
Flow chart of the real-time tracking program.

The function of the real-time tracking program is realized in DSP. In order to improve the real-time performance of the control program, the interrupt mode was adopted. The flow chart of the tracking program is shown in Figure 9.

The tracking program is mainly divided into 5 steps. The first step is interrupted by Timer 1 to enter the position control program and to read $\left(x_{\delta },y_{\delta }\right)$ and L. In the second step, according to the spot position value, enter into a different segment curve interval. In the third, according to the corresponding interval, calculate the parameters, including the gain $P_{L}$ of position loop, the number of command pulses N, and the command pulse frequency f. In the fourth, generate the control command pulse corresponding to the segment curve interval. In the fifth, complete a position control cycle and return to the main program.

## 5. Experimental Verification

### 5.1. Piecewise Low-Order Polynomial Least Squares Fitting

**Figure 10:**
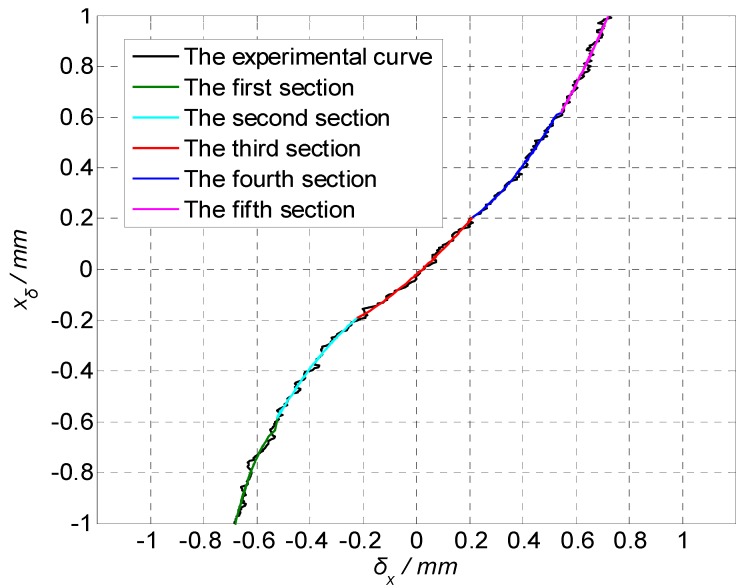
$\delta_{x}-x_{\delta }$ curve of the 4-QD in the X-direction.

The precision moving platform was used to move the 4-QD in the X-direction and Y-direction, respectively. Taking the X-direction as an example, the precision mobile platform feeds every 5 microns from −1 to 1 mm. By collecting the output voltage of the 4-QD, 400 sets of data were obtained and the curves $\delta_{x}-x_{\delta }$ are drawn as shown in Figure 10.

As can be seen from the experimental curve in Figure 10, there is an approximate linear relationship between the calculated value of the spot $\delta_{x}$ and the actual value $x_{\delta }$ of the spot in the area near the center of the detector. The closer to the center of the detector, the higher the detection accuracy. The farther away from the center of the detector, the greater the deviation.

The experimental data were fitted by piecewise low-order polynomial least squares fitting. The average absolute errors (MAE) and mean square errors (MSE) were calculated using different piecewise numbers and polynomial degrees. The results are shown in Table 1.

Generally speaking, if the number of segments is more and the polynomial degrees are higher, the fitting accuracy will be higher. However, more segments and higher polynomial degrees will lead to a great increase in the amount of calculation. Therefore, by analyzing and comparing the fitting accuracy, it can be seen that when the number of segments is greater than 4, increasing the number of fitting times cannot significantly improve the fitting accuracy, but will increase the burden of calculation. The contradiction between fitting accuracy and calculating speed can be well considered by using the least squares fitting of the 5-segment and the quadratic polynomial. The least squares fitting curve equation of the quadratic polynomial is:

(33) $$x_{\delta }=\{\begin{array}{l} -11.11849\delta_{x}^{2}-11.02477\delta_{x}-3.35345\;(\delta_{x}\leq -0.52)\\ -1.42177\delta_{x}^{2}-0.24315\delta_{x}-0.06751\;(-0.52<\delta_{x}\leq -0.22)\\ 0.68194\delta_{x}^{2}+0.92662\delta_{x}-0.02097\;(-0.22<\delta_{x}\leq 0.22)\\ 1.16339\delta_{x}^{2}+0.078\delta_{x}+0.11422\;(0.22<\delta_{x}\leq 0.52)\\ 1.60256\delta_{x}^{2}+0.13165\delta_{x}+0.07095\;(0.52\leq \delta_{x}) \end{array}.$$

### 5.2. Kalman Filtering

The four-way voltage $\left(U_{A}^{},U_{B}^{},U_{C}^{},U_{D}^{}\right)$ output by the 4-QD was converted to three-way voltages $\left(U_{I},U_{II},U_{III}\right)$ by the hardware conversion circuit, and their relationship is shown as follows:

(34) $$\{\begin{array}{l} U_{I}=(U_{A}+U_{D})-(U_{B}+U_{C})\\ U_{II}=(U_{A}+U_{B})-(U_{C}+U_{D})\\ U_{III}=U_{A}+U_{B}+U_{C}+U_{D} \end{array}.$$

The analog-digital sampling module was used to sample the output voltages of three channels every 0.1 ms, and a total of 100 groups of data were collected. 

**Figure 11:**
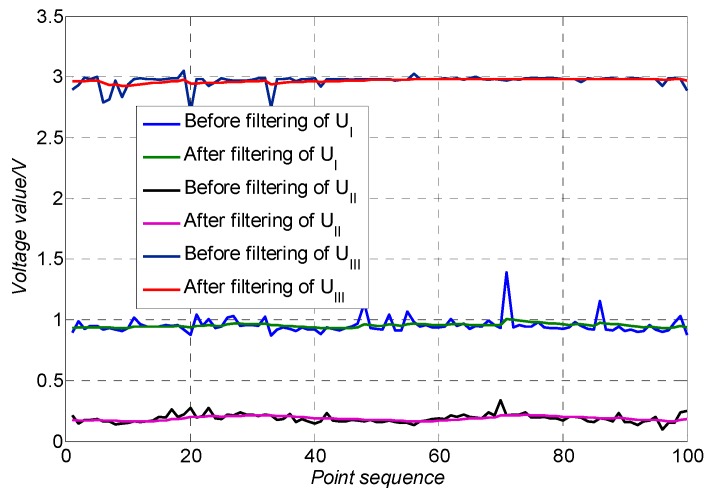
Output voltage filtering effect comparison.

The Kalman filtering algorithm was used to process the output voltages of the three channels, and the filtered data was obtained, which was compared with the data before filtering. The comparison results are shown in Figure 11. It can be seen from Figure 11, before the use of the Kalman filtering algorithm, the curve of three-way voltages fluctuates greatly, and after the use of the Kalman filtering algorithm, the noise of the voltages was greatly reduced. The maximum absolute error (MAE) and root mean square error (RMSE) are shown in Table 2. For the output voltage *U*_I_, the MAE and RMSE after filtering were reduced by 87% and 75%, respectively, and for *U*_II_, they reduced by 81% and 54%, respectively, and for *U*_III_, they reduced by 84% and 69%. It can be seen that the Kalman filter algorithm can effectively suppress the noise of the output voltages and improve the detection accuracy of the 4-QD significantly.

### 5.3. Dynamic Tracking Experiment

**Figure 12:**
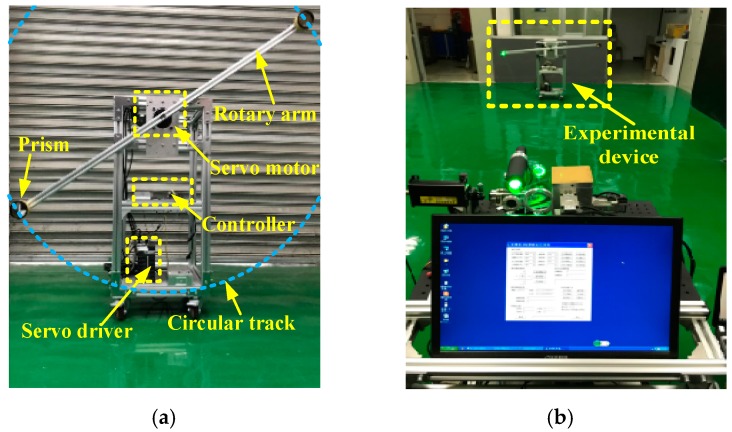
Rotary tracking test. (**a**) The experimental device; (**b**) Moving target tracking test.

In order to compare and analyze the performance of the traditional method and the improved method, a tracking test bed was built for the experiment of target tracking as shown in Figure 12. The rotary arm was driven by a servo motor, and a prism was installed at one end of the rotary arm. Theoretically, the rotation path of the prism was a circle with a diameter of 1.44 m.

In the experiment, the prism rotates at a certain speed. The LTPS tracks the prism and measures its trajectory. By comparison with the ideal circular track, the tracking accuracy was obtained. The maximum tracking speed (Smax) of the tracking system can be obtained by gradually increasing the speed of the rotating arm until the LTPS cannot track normally. The position error $\varepsilon_{i}$ in Equation (35) and root mean square error (RMSE) in Equation (36) were used to measure the accuracy of the system, as follows:

(35) $$\varepsilon_{i}^{}=\sqrt{{\left(x_{i}^{{}_{}}\right)}^{2}+{\left(z_{i}^{}\right)}^{2}}-720\;\left(i=1,2,\ldots ,n\right)$$

(36) $$RMSE=\sqrt{\frac{1}{n}\sum_{i=1}^{n}\varepsilon_{i}^{2}}$$

where $\left(x_{i},z_{i}\right)$ is the coordinate of the moving prism, and n is the number of points collected by the system.

The tracking experiments were carried out at the distance of 7.5, 15.5, and 23.5 m from the LTPS respectively. The Smax, $\varepsilon_{\max}$, and RMSE at different distances are shown in Table 3.

**Figure 13:**
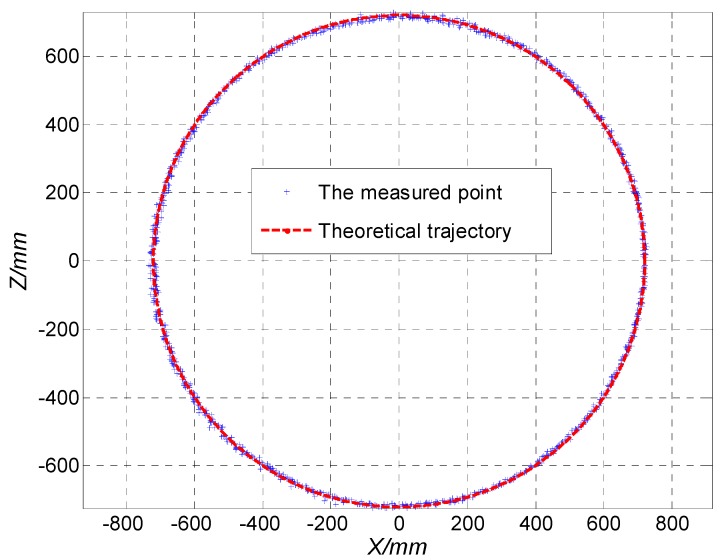
The tracking point of the rotating prism at 23.5 m.

The experimental results show that the maximum tracking distance of the traditional method is about 12 m, while the improved method is more than 30 m. When the tracking distance exceeds 12 m, the traditional method completely fails. Compared with the tracking speed and tracking accuracy of the traditional method and the improved method, it can be seen that at 7.5 m, the maximum tracking speed was not significantly improved after the improved method, but the tracking accuracy was improved obviously. Although the Smax of both methods can reach about 2 m/s, the $\varepsilon_{\max}$ of the target trajectory was reduced by 13.4% and the RMSE was reduced by 32.4% after using the improved method. This is because the improved method of piecewise low-degree polynomial fitting and the Kalman filter increases the calculation burden while improving the detection accuracy. As a result, the maximum tracking speed was not significantly improved, and the tracking accuracy was improved. By comparing the tracking effect of the improved method at 15 and 25 m, we can see that with the increase of the target distance, the maximum tracking speed and tracking accuracy gradually decrease. The reason is that, for a long distance, the divergence of the indicator laser becomes more and more serious, which will reduce the signal-to-noise ratio of the 4-QD and the accuracy of the spot position detection, and ultimately leads to the decrease of accuracy and speed. However, due to the improved method, the Smax can still reach 1.13 m/s at 23.5 m. The tracking point of the rotating prism at 23.5 m measured by the system is shown in Figure 13, and the RMSE error is less than 5.71 mm.

## 6. Conclusions

In this paper, a tracking and positioning system for a moving target was constructed, and a mathematical model of tracking and positioning was established. Aiming at the problem of low precision and poor real-time performance of traditional methods, an improved method of spot position detection based on a piecewise low-order polynomial least squares fitting algorithm and a Kalman filter algorithm was proposed. For the collected experimental data of the spot position, the detection curve of the spot position was established using a 5-segment quadratic polynomial least squares fitting. Aiming at the problem of high noise and low sampling accuracy of output voltages of the detector, a Kalman filter algorithm was proposed to improve the detection accuracy. The system experiment platform and rotation tracking test bed were established, and the tracking experiment was completed. The experimental results of the traditional method and the improved method were compared and analyzed. The results show that the polynomial fitting with the 5-segment and quadratic polynomial can not only meet the fitting accuracy, but also has a faster calculation speed, which is suitable for real-time processing by a microprocessor. A Kalman filter can significantly reduce the noise of 4-QD output voltages and improve the detection accuracy. The experimental results of rotation tracking show that the maximum tracking distance of the system was increased from 12 m to more than 30 m. At 23.5 m, the maximum tracking speed was 1.13 m/s, and the RMSE was less than 5.71 mm. Therefore, the improved method based on the 5-segment quadratic polynomial least squares fitting and the Kalman filter can effectively remedy the shortcomings of traditional methods, improve the accuracy of spot detection, and significantly improve the performance of the laser tracking and positioning system.

## Figures and Tables

**Table 1:** Fitting errors of experimental curves. MAE: average absolute errors; MSE: mean square errors.

	2 Sections		3 Sections		4 Sections		5 Sections	
	MAE	MSE	MAE	MSE	MAE	MSE	MAE	MSE
**One Degree Polynomial**	0.1424	0.0035	0.0804	0.0008	0.0894	0.0006	0.0899	0.0005
**Quadratic Polynomial**	0.0821	0.0005	0.0866	0.0004	0.0839	0.0004	0.0754	0.0003
**Cubic Polynomial**	0.0860	0.0004	0.0826	0.0004	0.0745	0.0003	0.0754	0.0003
**Quartic Polynomial**	0.0855	0.0004	0.0781	0.0004	0.0751	0.0003	0.0733	0.0003

**Table 2:** Comparison of output voltage results. MAE: maximum absolute error; RMSE: root mean square error.

	*U*_I_		*U*_II_		*U*_III_	
	MAE	RMSE	MAE	RMSE	MAE	RMSE
**Before Filtering**	0.434	0.064	0.148	0.035	0.257	0.049
**After Filtering**	0.055	0.016	0.028	0.016	0.042	0.015

**Table 3:** Tracking experimental results.

	Traditional Method			Improved Method		
Tracking distance (m)	7.5	15.5	23.5	7.5	15.5	23.5
S_max_ (m/s)	1.96	——	——	2.11	2.04	1.13
$\varepsilon_{\max}$ (mm)	16.25	——	——	14.07	15.90	21.23
RMSE (mm)	6.79	——	——	4.59	5.42	5.71

## References

[1] Nelson W., Palastro J.P., Wu C., Davis C.C. (2016). Using an incoherent target return to adaptively focus through atmospheric turbulence. Opt. Lett..

[2] Yu J., Li Q., Li H., Wang Q., Zhou G.Z., He D., Xu S.X., Xia Y.X., Huang Y.M. (2019). High-precision light spot position detection in low SNR condition based on quadrant detector. Appl. Sci..

[3] Li Q., Xu S., Yu J., Yan L., Huang Y. (2019). An improved method for the position detection of a quadrant detector for free space optical communication. Sensors.

[4] Hu C., An R., Zhang C., Lei H., Hu X., Li H., Hu X. (2015). Design of a high-quality optical conjugate structure in optical tweezers. Appl. Opt..

[5] Kuang C., Hong E., Feng Q.B., Zhang B., Zhang Z.F. (2007). A novel method to enhance the sensitivity for two-degrees-of-freedom straightuess measurement. Meas. Sci. Technol..

[6] Kuang C., Hong E., Ni J. (2007). A high-precision five-degree-of-freedom measurement system based on laser collimator and interferometry techniques. Rev. Sci. Instrum..

[7] Li K., Kuang C., Liu X. (2013). Small angular displacement measurement based on an autocollimator and a common-path compensation principle. Rev. Sci. Instrum..

[8] Liu C., Fang D., Yang Z., Jiang H.B., Chen X.J., Wang W., Xing T.Z., Cai L. (2015). RSS distribution-based passive localization and its application in sensor networks. IEEE Trans. Wirel. Commun..

[9] Gao M., Yu M., Guo H., Xu Y. (2019). Mobile robot indoor positioning based on a combination of visual and inertial sensors. Sensors.

[10] Chen M., Yang Y., Jia X., Gao H.Y. (2013). Investigation of positioning algorithm and method for increasing the linear measurement range for four-quadrant detector. Optik.

[11] Vo Q., Zhang X., Fang F. (2019). Extended the linear measurement range of four-quadrant detector by using modified polynomial fitting algorithm in micro-displacement measuring system. Opt. Laser Technol..

[12] Salmanpour A., Nejad S.M. (2011). The performance improvement of the target position determining system in laser tracking based on 4q detector using neural network. World Acad. Sci. Eng. Technol..

[13] Lee E.J., Park Y., Kim C.S., Kouh T. (2010). Detection sensitivity of the optical beam deflection method characterized with the optical spot size on the detector. Curr. Appl. Phys..

[14] Lu C., Zhai Y.S., Wang X.J., Guo Y.Y., Du Y.X., Yang G.S. (2014). A novel method to improve detecting sensitivity of quadrant detector. Opt. Int. J. Light Electron Opt..

[15] Wu J., Zhao B., Wu Z. Improved measurement accuracy of the spot position on an InGaAs quadrant detector by introducing Boltzmann function. Proceedings of the 2015 International Conference on Optoelectronics and Microelectronics (ICOM).

[16] Xiao F. (2019). Multi-sensor data fusion based on the belief divergence measure of evidences and the belief entropy. Inf. Fusion.

[17] Xiao F., Qin B. (2018). A weighted combination method for conflicting evidence in multi-sensor data. Fusion. Sens..

[18] Zhang W., Guo W., Zhang C., Zhao S. (2019). An online calibration method for a galvanometric system based on wavelet kernel ELM. Sensors.

[19] Makynen A.J., Kostamovaara J.T., Myllyla R.A. (1995). A high-resolution lateral displacement sensing method using active illumination of a cooperative target and a focused four-quadrant position-sensitive detector. IEEE Trans. Instrum. Meas..

[20] Manojlovic L.M., Barbaric Z.P. (2009). Optimization of optical receiver parameters for pulsed laser-tracking systems. IEEE Trans. Instrum. Meas..

[21] Zhao X., Tong S., Jiang H. (2010). Experimental testing on characteristics of four-quadrant detector. Opt. Precis. Eng..

[22] Tang Y.Q., Gu G.G., Qian W.X., Chen Q., Zhang J. (2017). Laser spot center position algorithm of four-quadrant detector based on Gaussian distribution. Infrared Laser Eng..

[23] Maybeck P.S., Cox I.J., Wilfong G.T. (1990). The Kalman Filter: An Introduction to Concepts. Autonomous Robot Vehicles.

[24] Kawase T., Tsurunosono H., Ehara N., Sasase I. (2001). A Kalman tracker with a turning acceleration estimator. Electron. Commun. Jpn..

